# Supplementary material for: Modular reactor for in situ X-ray scattering, spectroscopy and ATR-IR studies of solvothermal nanoparticle synthesis
Source: J Synchrotron Radiat. 2026 Jan 1;33(Pt 1):142–53. doi: 10.1107/S1600577525009634 (PMC12809455; doi:10.1107/S1600577525009634)
Supplement: Supplementary file 1 [file s-33-00142-sup1.pdf]

JOURNAL OF SYNCHROTRON RADIATION

Volume 33 (2026)

Supporting information for article:

Modular reactor for *in situ* X-ray scattering, spectroscopy and ATR-IR studies of solvothermal nanoparticle synthesis

Dorota Koziej, Sani Y. Harouna-Mayer, Melike Gumus Akcaalan, Jagadesh Kopula Kesavan, Tjark R. L. Groene, Lars Klemeyer, Sarah-Alexandra Hussak, Lukas Grote, Davide Derelli, Francesco Caddeo, Cecilia Zito, Paul Stützle, Dorota Speer, Ann-Christin Dippel, Blanka Detlefs, Yannik Appiarius, Axel Jacobi von Wangelin and Dorota Koziej

The overview of present and previously reported reactor designs, along with a comparison of their heating performance, durability, and cost, is shown in Table S1. The heating rate is one of the most critical parameters for achieving controlled reaction conditions. The PEEK and all-glass inlets show almost identical heating profiles and only slight differences in cooling profiles. For scattering applications using a glass vial embedded in a PEEK holder, as employed in (Derelli et al., 2024), heat transfer to the solvent is somewhat reduced due to the insulating air gap between the two materials.

The PEEK and all-glass inlets have the same volume of 1.25 ± 0.03 mL. The volume deviation is primarily determined by the precision of the machining. The reaction solution is continuously stirred, further ensuring the homogeneity of both the precursor solution and the temperature.

Regarding durability, the metal housing provides comparable mechanical stability across all designs, while the glass components are inherently more fragile than PEEK. However, the all-glass inlet, owing to its greater wall thickness, is considerably more robust than the thin-walled glass vials used in other setups, while remaining suitable for scattering experiments.

The primary cost of an inlet arises from material expenses, with PEEK being more expensive (6 Euro/inlet) than borosilicate glass (10 cents/inlet). Nevertheless, the overall contribution of to the total cost remains modest due to the limited material quantity used and its reusability. All components of the cell were machined within a few working days. The machining of the inlets takes up to 2 hours, where most of the time is needed for setting up the machine.

Overall, our reactor meets the requirements for a versatile, durable, and cost-efficient *in situ* design, providing stable thermal performance and flexible inlet options for a wide range of synchrotron-based experiments.

Table S1 Comparison of present and previous reactor designs.

	Reference	Materials	Heating element number/position relating to the heating condition	Durability
1	(Staniuk *et al.*, 2014)	PEEK inlet in Brass housing	Two heating elements connected to the housing from bottom	High
2	(Grote *et al.*, 2021)	PEEK inlet in Brass housing	Two heating elements connected to the housing from bottom	High
3	(Derelli *et al.*, 2024)	Glass vial+PEEK inlet in Brass housing	Two heating elements embedded in the housing	Low
4	(Klemeyer *et al.*, 2024, 2025)	Glass vial+PEEK inlet in Aluminium housing	Four heating elements embedded in the housing	Medium
5	(Harouna-Mayer *et al.*, 2025)	Glass vial+PEEK inlet in Aluminium housing	Four heating elements embedded in the housing	Medium
6	Present work	- PEEK inlet<br>- Glass inlet<br>- Glass vial+Copper inlet in Aluminium housing	Four heating elements embedded in the housing	- High<br>- Medium<br>- Medium/Low

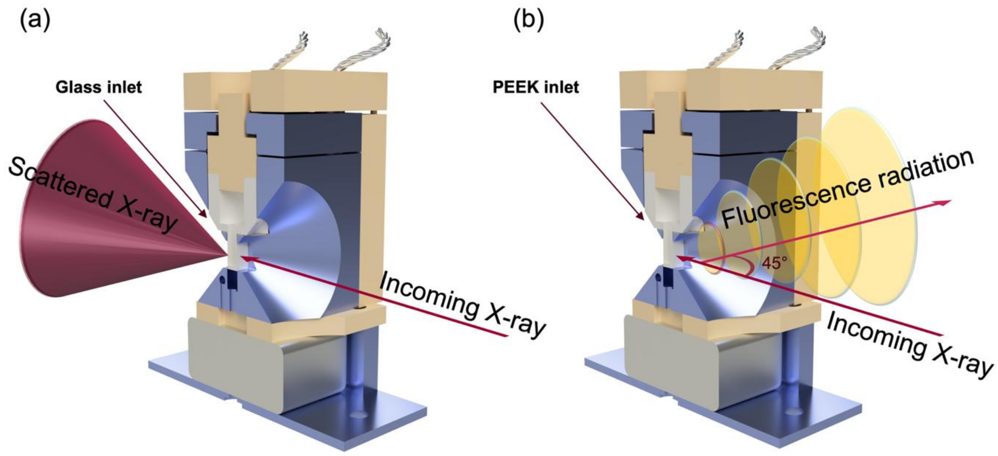

Figure S1 Cross section of the reactor including the x-ray beam path. *(a)* Transmission geometry used for scattering experiments. *(b)* Reflection geometry used for HERFD-XAS measurements.

Table S2 Instrumentally achievable maximum q-values ($q_{max,inst}$) at different x-ray energies and sample-to-detector distances (SDD), assuming a quadratic 2D area detector with a height (DH) of 500 mm and the primary x-ray beam centered on the detector. Only the detector region with full azimuthal coverage is considered, *i.e.* the corners are excluded. $q_{max,inst}$ is defined by the maximum scattering angle $2\Theta_{max,inst}$ as $q_{max,inst} = \frac{4\pi}{\lambda}\sin(\frac{2\Theta_{max,inst}}{2})$, where λ is the x-ray wavelength. At low SDD and large DH, $2\Theta_{max,inst}$ equals the reactor opening angle (45° in our case), otherwise it is determined by the SDD and DH: $2\Theta = \arctan(\frac{DH/2}{SDD})$. We calculate $q_{max,inst}$ values for x-ray energies of 5, 10, 20, 50, and 100 keV, and for SDDs of 250, 500, 1500, and 3000 mm. At an SDD of 250 mm, $2\Theta_{max,inst}$ equals 45° based on the above considerations. For shorter SDDs, $q_{max,inst}$ would be limited by the reactor opening angle.

Energy (keV)	λ (Å)	SDD (mm)	$2\Theta_{max,inst}$ (°)	$q_{max,inst}$ (Å^{-1})
5	2.480	250	45.0	1.9
		500	26.9	1.2
		1500	9.5	0.4
		3000	4.8	0.2
10	1.240	250	45.0	3.9
		500	26.9	2.3
		1500	9.5	0.8
		3000	4.8	0.4
20	0.620	250	45.0	7.8
		500	26.9	4.7
		1500	9.5	1.7
		3000	4.8	0.8
50	0.248	250	45.0	19.4
		500	26.9	11.6
		1500	9.5	4.2
		3000	4.8	2.1
100	0.124	250	45.0	38.8
		500	26.9	23.3
		1500	9.5	8.4
		3000	4.8	4.2

The reactor was adapted to the cooling application and used with an injection cap for reactions operating at sub-zero temperatures and under inert conditions. For the updated version, the essential modification was to incorporate the Peltier element (Adaptive-ETC-128-14-06-E) into the design to cool the system temperature down to -20 °C. The cooling places further requirements, like cooling the hot side of the Peltier element to avoid overheating and achieve better performance. This requirement is met by placing water-cooled copper blocks next to the Peltier elements by taking advantage of copper's excellent thermal conductivity. The water cooling that cycles inside the copper blocks was provided with a chiller (Hei-Chill series- Heidolph) set at 5 °C. As in the previous design, the reactor temperature was monitored using a sensor mounted on the brass body, whereas the temperature of the Peltier element was controlled by a thermoelectric cooler (TEC) (Meerstetter Engineering TEC-1167) through a side-mounted sensor embedded in the copper block.

In terms of thermal insulation, polypropylene (PP) insulators are positioned on the front, back, and top sides of the brass housing to decrease the thermal leakage from the system to the air.

As for the inlet preference, both glass and PEEK inlets can be used in the reactor. An N_2 connection, which is embedded in the housing, blows vertically through the inlet's X-ray window. Thereby, possible frost that can negatively affect the measurement is prevented by displacing the air moisture from the window surface. Figure S2 presents the configuration where the Peltier cooling-incorporated reactor is used with the glass inlet equipped with a brass injection cap.

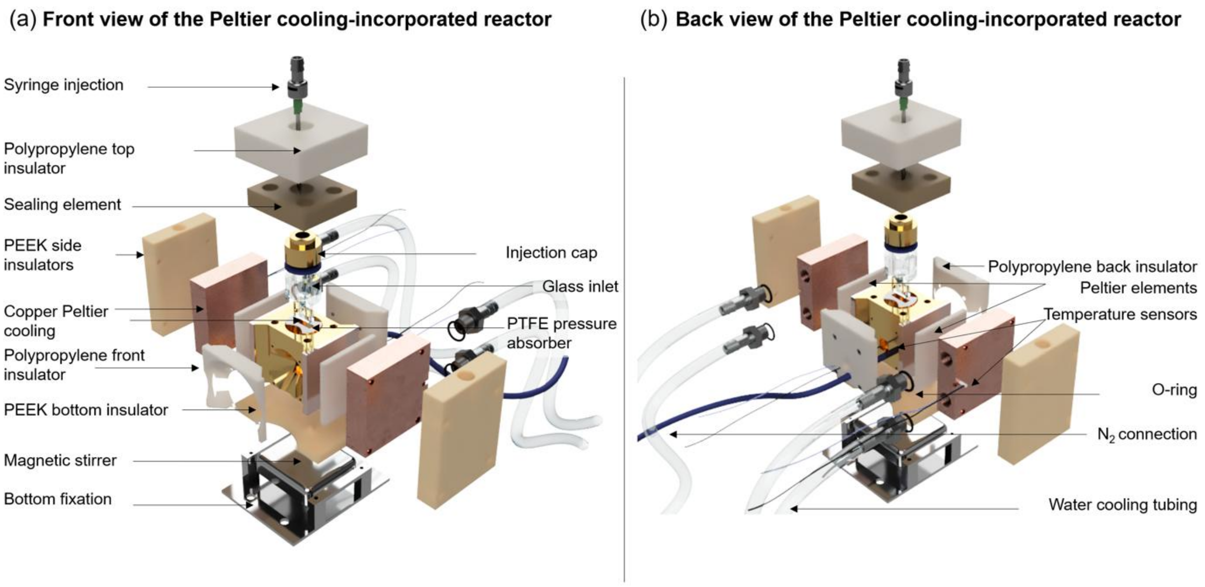

Figure S2 The rendered image of the Peltier cooling-incorporated reactor. *(a)* Exploded front view. *(b)* Exploded back view.

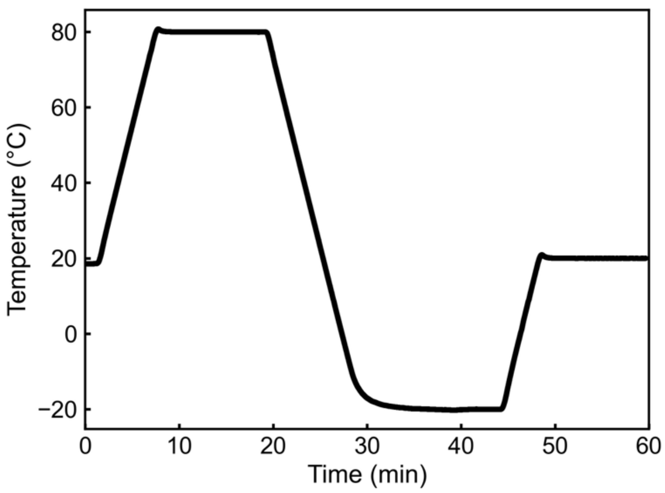

Figure S3 Temperature profile of the reactor equipped with Peltier cooling, showing the heating ramp to 80 °C followed by active cooling to -20 °C.

As mentioned earlier, the IR-adapted cap can be used with an ATR-IR fiber cable, allowing the cable tip to contact the solution directly and get adequate data. The IR cap consists of two components, a hollow brass screw fastener and a hollow PEEK component. The fiber cable passes through the hollow brass fastener and is screwed to the hollow PEEK cap, and these pieces are sealed into the inlet via an O-ring. Similarly, the pressure sensor is also threaded into a hollow PEEK cap, allowing the sensor to measure the pressure close to the solution interface. The pressure sensor head is wrapped in PTFE tape first to avoid any loose connection that can affect the pressure measurement. Then, it is screwed to the cap and sealed through an O-ring to the inlet.

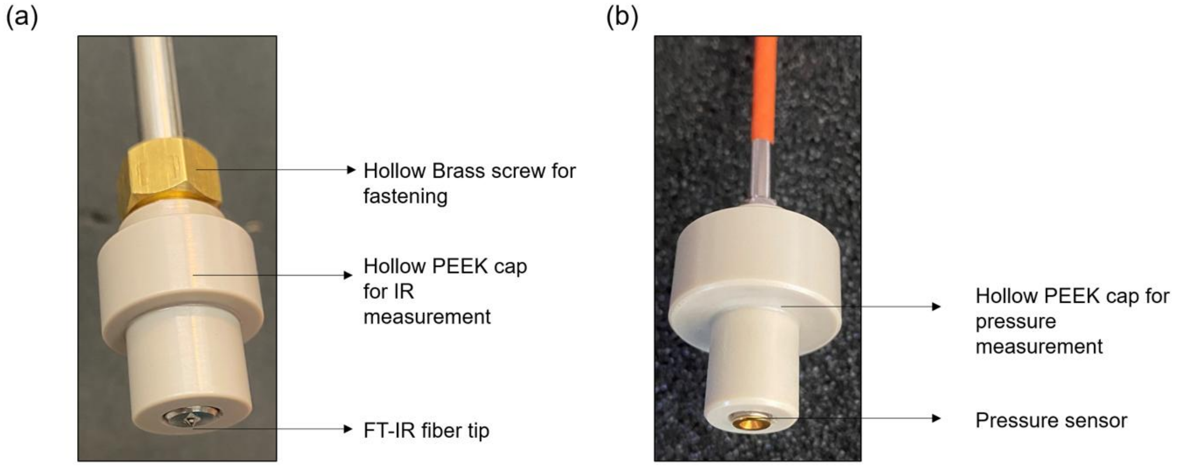

Figure S4 *(a)* The picture of Brass + PEEK cap equipped with an optical ATR-FTIR fiber probe. *(b)* The picture of a PEEK cap with a pressure sensor.

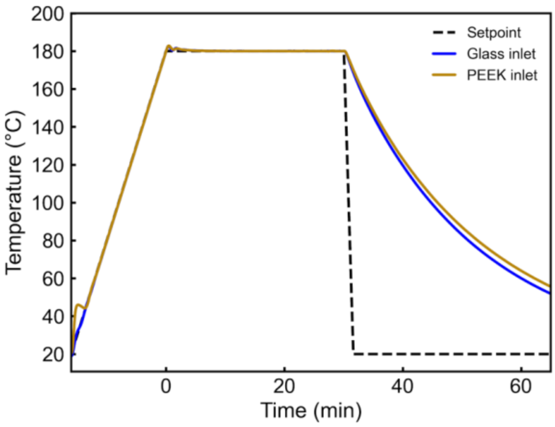

Figure S5 Measured temperature profiles of the reactor during heating of BnOH to 180 °C in (*a*) a glass and (*b*) a PEEK inlet. The setpoint represents the programmed heating profile of the controller, which regulates the heating.

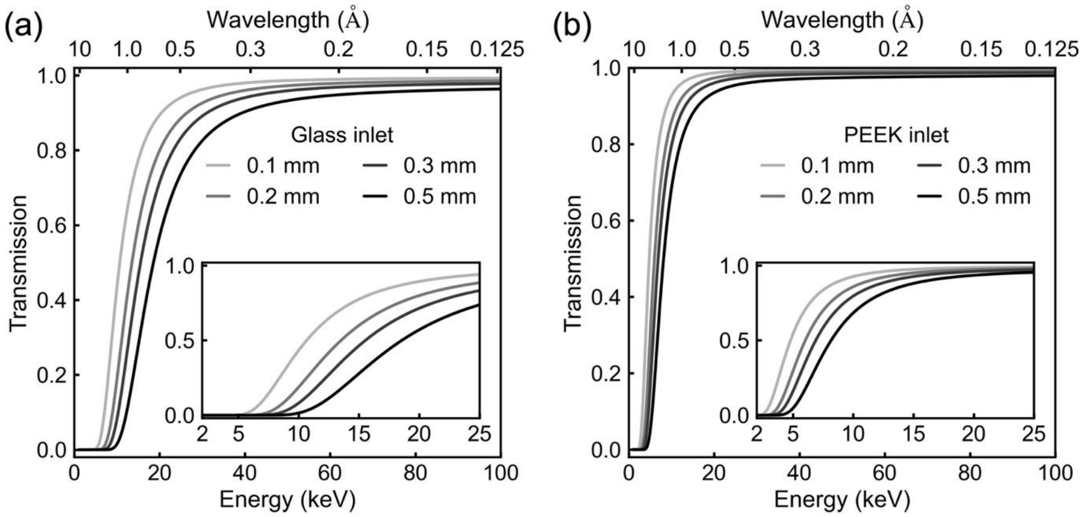

Figure S6 X-ray transmission of (*a*) the glass and (*b*) PEEK inlet at wall thicknesses between 0.1 and 0.5 mm. The inlet shows a zoom between 2 and 25 keV x-ray energy.

Table S3 Refined structural parameters from Rietveld analysis of the final stage of the reaction.

R_{wp} (%)	4.34
a (Å)	8.449
Size (nm)	4.3
Fe^{3+} U_{iso} (Å^2)	0.0130
Fe^{2+} U_{iso} (Å^2)	0.0096
O^{2-} U_{iso} (Å^2)	0.0066

Table S4 Refined structural parameters from PDF analysis for the initial, intermediate, and final stage of the reaction.

	Reaction stage	Initial stage	Intermediate stage	Final stage
	Reaction time	-15 min	5 min	75 min
	R_W (%)	43.9	19.0	19.8
$Fe(acac)_3$	Relative scale	1.000	1.000	0.000
	δ_2 (Å^2)	1.591	1.591	-
	Fe U_{iso} (Å^2)	9.530 x10^{-4}	2.077 x10^{-4}	-
	O U_{iso} (Å^2)	1.506 x10^{-2}	1.000 x10^{-1}	-
	C_1 U_{iso} (Å^2)	3.354 x10^{-2}	9.211 x10^{-3}	-
	C_2 U_{iso} (Å^2)	1.000 x10^{-1}	2.165 x10^{-2}	-
	C_3 U_{iso} (Å^2)	1.966 x10^{-3}	9.961 x10^{-2}	-
$[Fe_3O(AcO)_6(H_2O)_3]^+$	Relative scale	-	0.922	-
	δ_2 (Å^2)	-	3.369	-
	Fe U_{iso} (Å^2)	-	1.365 x10^{-2}	-
	O U_{iso} (Å^2)	-	1.000 x10^{-1}	-
	C U_{iso} (Å^2)	-	3.399 x10^{-2}	-
Fe_3O_4	Relative scale	0.000	0.703	1.000
	a (Å)	-	8.859	8.437
	δ_2 (Å^2)	-	2.370	0.823
	Fe U_{11} (Å^2)	-	1.783 x10^{-1}	8.782 x10^{-3}
	Fe U_{12} (Å^2)	-	5.192 x10^{-4}	1.645 x10^{-3}
	Fe U_{iso} (Å^2)	-	6.333 x10^{-3}	2.466 x10^{-3}
	O U_{11} (Å^2)	-	2.610 x10^{-1}	1.622 x10^{-2}
	O U_{12} (Å^2)	-	2.626 x10^{-2}	1.413 x10^{-2}

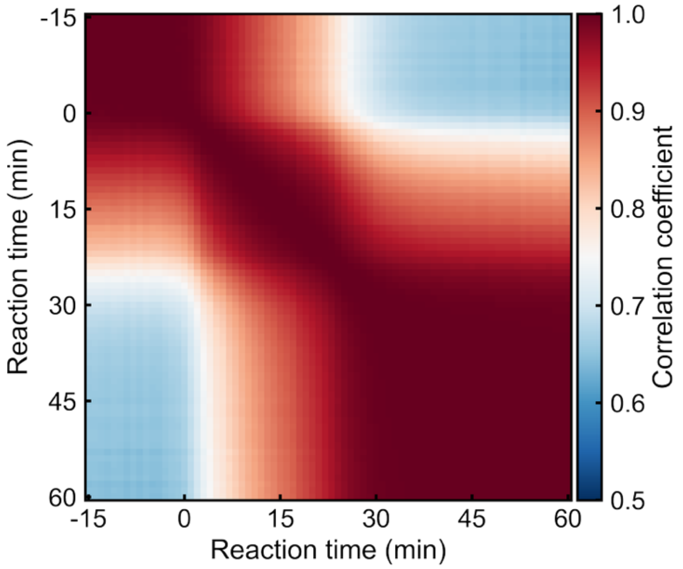

Figure S7 Pearson correlation map of the *in situ* PXRD data. Pearson correlation mapping quantifies the similarity between diffraction patterns collected at different time points, providing a visual representation of structural evolution during the reaction. Correlation coefficient values close to 1 (red) indicate high similarity, whereas low correlations (blue) indicate strong differences. The maps clearly reveal three distinct reaction stages corresponding to the precursor complex, the intermediate phase, and the final Fe_3O_4 product. The first transition occurs shortly after 0 min, and the second around 25 min as indicated by the nodes along the diagonal. The Pearson correlation map was calculated for PXRD pattern between q_{min} of 1.9 Å^{-1} and q_{max} of 25.0 Å^{-1}.

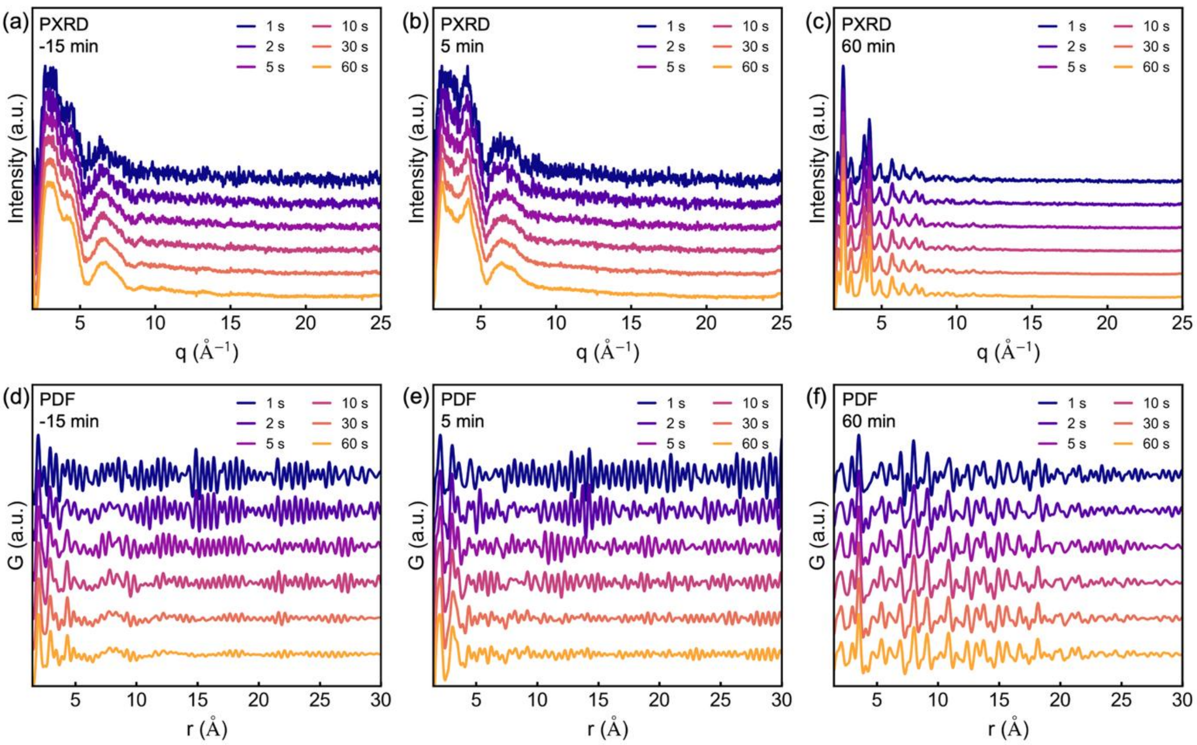

Figure S8 PXRD and PDF data collected at –15, 5, and 60 minutes reaction time. Data are averaged over 1 to 60 individual patterns, each acquired with a 1 s exposure time.

Table S5 Assigned vibrational modes observed in the *in situ* ATR-FTIR spectra.

Peak position (cm^{-1})	Vibrational mode	Assigned to
1739	ν(C=O)	Benzyl acetate
1717	ν(C=O)	Acetone
1380	$\delta_{as}(CH_3)$	Methyl
1361	$\delta_{s}(CH_3)$	Methyl
1225	ν(C–O)	Benzyl acetate

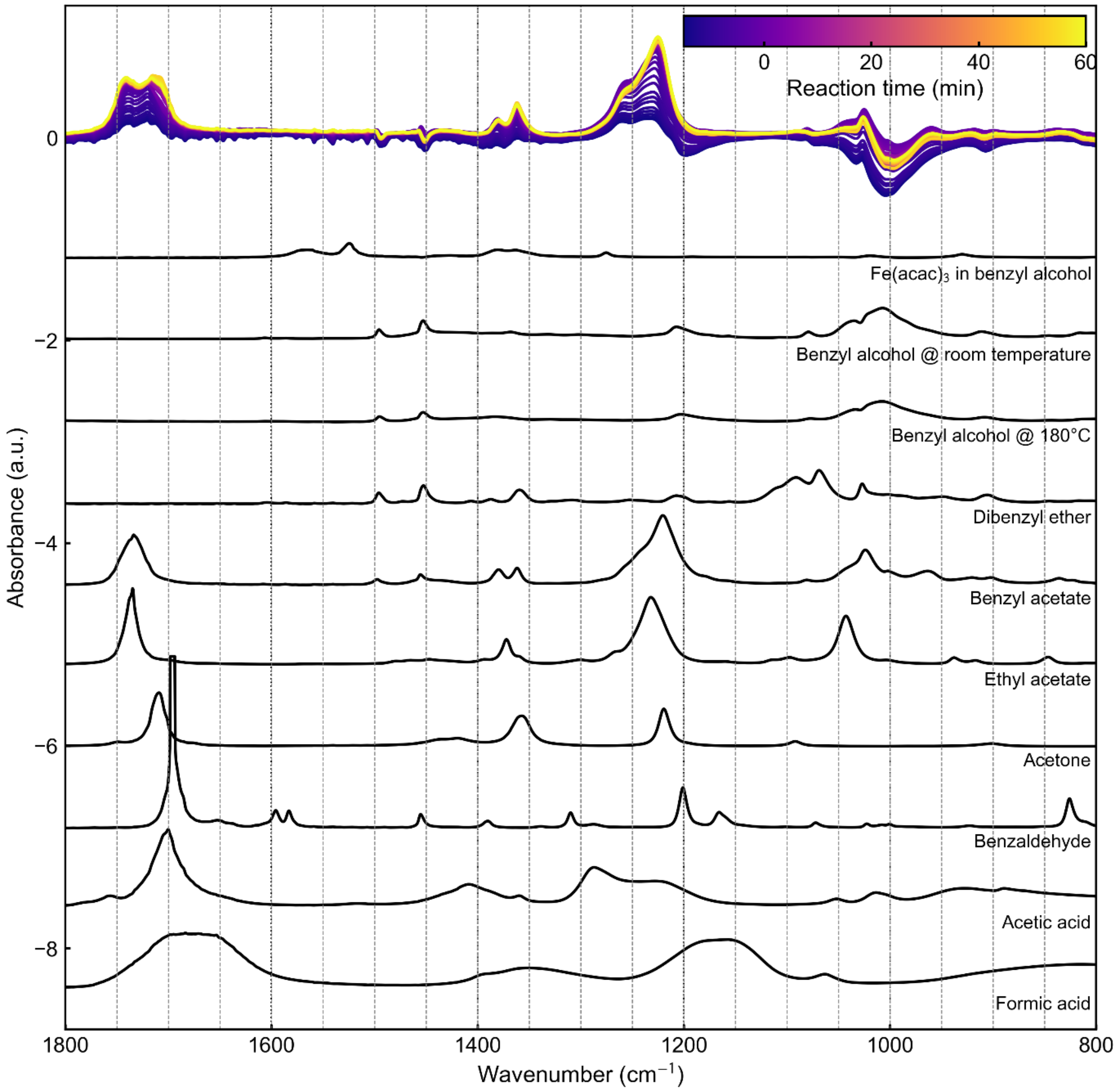

Figure S9 *In situ* ATR-FTIR analysis of the reaction of $Fe(acac)_3$ to Fe_3O_4 in BnOH, compared to reference spectra of $Fe(acac)_3$ dissolved in BnOH, BnOH at room temperature, and at 180 °C, dibenzyl ether, benzyl acetate, ethyl acetate, acetone, benzaldehyde, acetic acid, and formic acid.

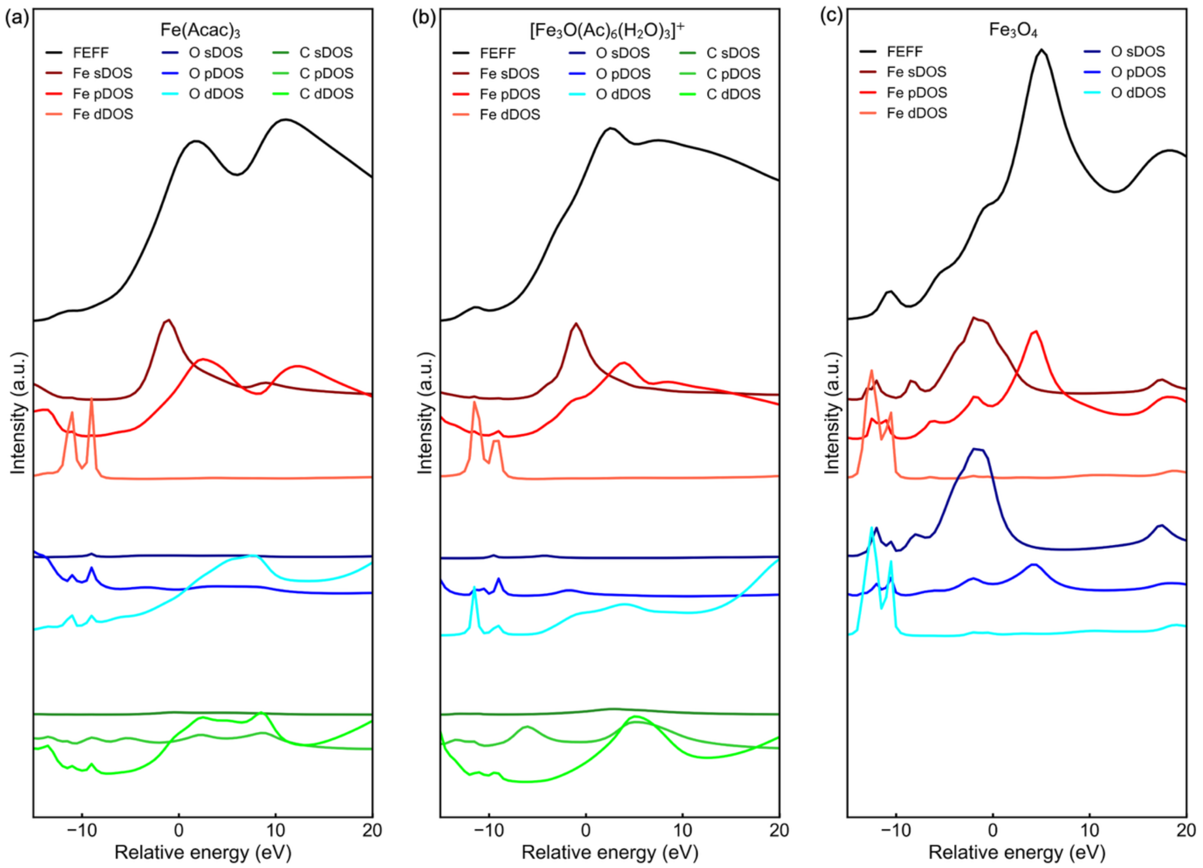

Figure S10 FEFF simulated XANES spectra and their corresponding density of states (DOS) of s, p and d states of (*a*) Fe(acac)$_3$, (*b*) [$Fe_3O(AcO)_6(H_2O)_3$]$^+$, and (*c*) Fe_3O_4.

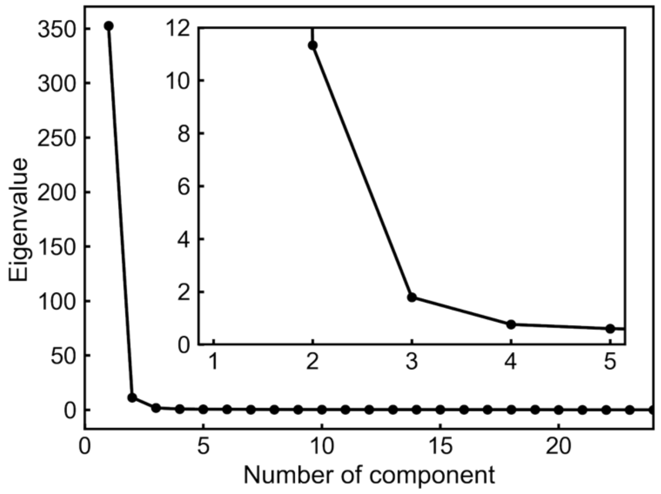

Figure S11 Eigenvalue profile of MCR-ALS analysis.

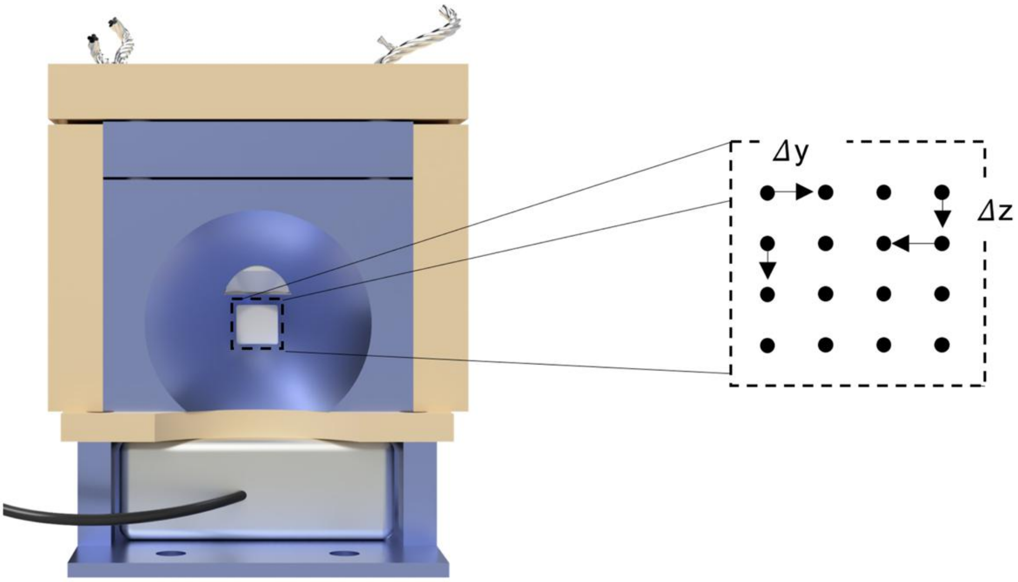

Figure S12 Schematic of the scanning pattern across the x-ray window used for XAS measurements to minimize beam damage. Repeated exposure of the same sample area is avoided by collecting spectra at different sample positions along a defined trajectory with step sizes Δy and Δz along the horizontal and vertical axes of the sample window.

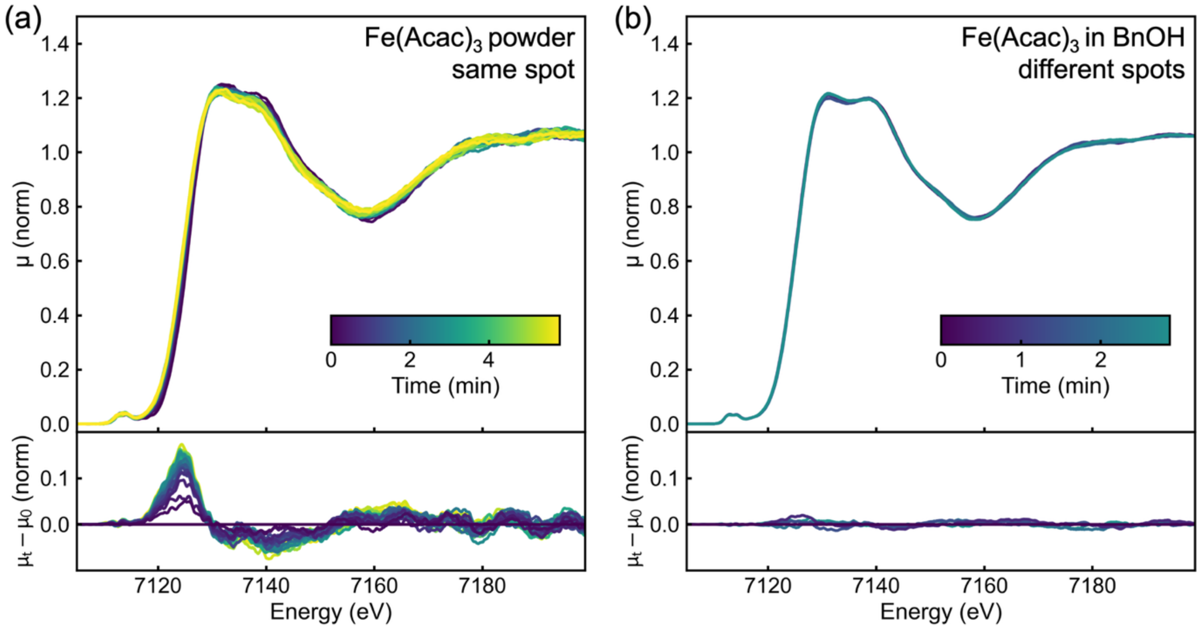

Figure S13 Beam damage study on $Fe(acac)_3$. (*a*) Fe K-edge HERFD-XANES spectra of $Fe(acac)_3$ powder recorded at room temperature over 30 consecutive scans (12 s each) at the same sample position on the sample. (*b*) Fe K-edge HERFD-XANES data of $Fe(acac)_3$ in benzyl alcohol (BnOH) recorded at room temperature over 5 consecutive scans (42s each) at different sample positions. The upper panels in (*a*) and (*b*) show the HERFD-XANES spectra while the lower panels show the difference of the spectra at the respective time μ_t and the first spectra at 0 min μ_0. The spectra recorded at the same

sample spot (*a*) show beam damage evident by the shift of the absorption edge and the decrease of the white-line intensity. The spectra recorded at different sample spots (*b*) show no strong spectral changes. This demonstrates that scanning across different sample positions, as illustrated in Figure S12, effectively enables avoiding beam damage. These scanning conditions were used for the *in situ* Fe K-edge HERFD-XANES measurements.

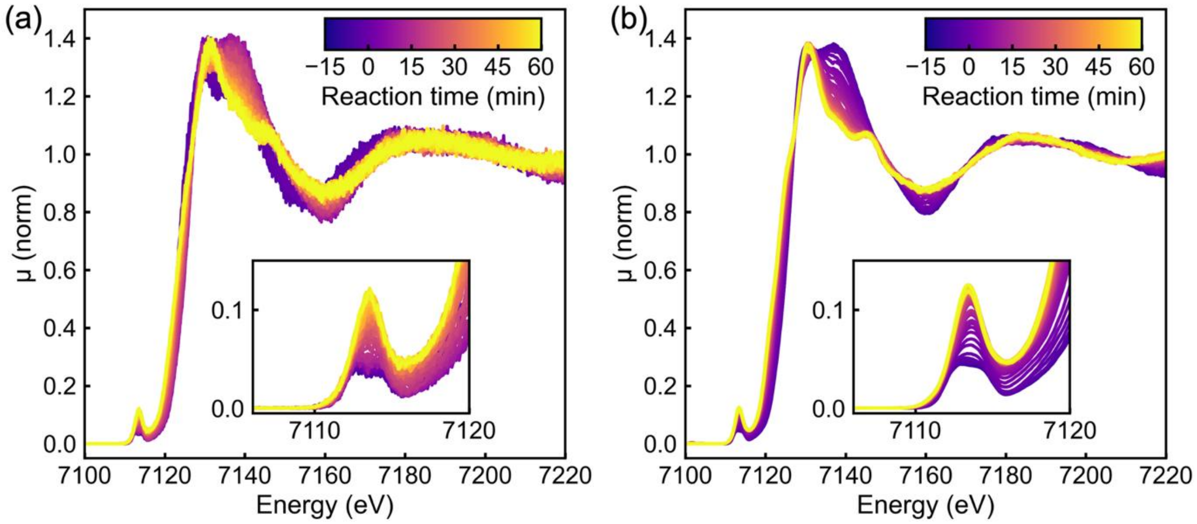

Figure S14 *In situ* Fe K-edge HERFD-XANES data before (*a*) and after (*b*) processing as discussed in the experimental section.
